# The effect of isoflurane on ^18^F-FDG uptake in the rat brain: a fully conscious dynamic PET study using motion compensation

**DOI:** 10.1186/s13550-016-0242-3

**Published:** 2016-11-25

**Authors:** Matthew G. Spangler-Bickell, Bart de Laat, Roger Fulton, Guy Bormans, Johan Nuyts

**Affiliations:** 1 Department of Imaging and Pathology, KU Leuven - University of Leuven, Nuclear Medicine & Molecular Imaging, Medical Imaging Research Center (MIRC), Leuven, Belgium; 2 Brain & Mind Centre and the Faculty of Health Sciences, University of Sydney, Sydney, Australia; 3 Department of Nuclear Medicine, Westmead Hospital, Sydney, Australia; 4 Department of Radiopharmacy, KU Leuven, Leuven, Belgium

**Keywords:** PET, Preclinical, Motion compensation, FDG, Anaesthesia

## Abstract

**Background:**

In preclinical positron emission tomography (PET) studies an anaesthetic is used to ensure that the animal does not move during the scan. However, anaesthesia may have confounding effects on the drug or tracer kinetics under study, and the nature of these effects is usually not known.

**Method:**

We have implemented a protocol for tracking the rigid motion of the head of a fully conscious rat during a PET scan and performing a motion compensated list-mode reconstruction of the data. Using this technique we have conducted eight rat studies to investigate the effect of isoflurane on the uptake of ^18^F-FDG in the brain, by comparing conscious and unconscious scans.

**Results:**

Our results indicate that isoflurane significantly decreases the whole brain uptake, as well as decreasing the relative regional FDG uptake in the cortex, diencephalon, and inferior colliculi, while increasing it in the vestibular nuclei. No statistically significant changes in FDG uptake were observed in the cerebellum and striata.

**Conclusion:**

The applied event-based motion compensation technique allowed for the investigation of the effect of isoflurane on FDG uptake in the rat brain using fully awake and unrestrained rats, scanned dynamically from the moment of injection. A significant effect of the anaesthesia was observed in various regions of the brain.

## Background

In preclinical positron emission tomography (PET) studies, an anaesthetic, such as isoflurane, ketamine or chloral hydrate, is usually used to ensure that the animal remains motionless for the duration of the scan. The effect of the anaesthetic on the kinetics of the drug or radioactive tracer under study is not always known and may confound the results of the investigation. This is especially problematic for translational studies since anaesthesia is usually avoided in the clinic. A summary of reports on the effect of anaesthetics in preclinical studies is given in [1, 2]. In general, three methods are used preclinically to establish the effect of an anaesthetic. The first is to compare the findings of similar investigations using different anaesthetics [3]; however, inferring the individual effects of the anaesthetics separately is complex and difficult to verify. The second method is to infuse the animals with the tracer or drug while conscious and then, after some time, anaesthetise and scan them, thus acquiring a static scan where the tracer or drug metabolism is not affected by the anaesthetic [4]; this assumes that the tracer washout is negligible. However, to fully quantify the effect of anaesthesia on the tracer kinetics, a dynamic scan starting from the time of injection is necessary. The third method, which does allow for dynamic scanning, is to physically restrain the animals (particularly the head for brain imaging) such that motion is impossible [5]. In this case though, the effect of stress on the kinetics is another confounding factor which is difficult to quantify and is known to affect brain function in many cases [6, 7]. A novel method for conducting brain scanning of conscious animals is the so-called RatCAP [8, 9], where a miniaturised PET scanner is surgically mounted directly onto the head of a rat and thus moves rigidly with the brain, avoiding motion between the brain and the scanner. While this system is promising, it has a lower sensitivity than commercial scanners and may inhibit the natural movement of the rat, possibly inducing stress in the animal.

As stated in [2], a study involving fully awake, unrestrained animals would be ideal for quantifying the effect of an anaesthetic. Several groups, including our own, have been conducting research into tracking the head motion of an awake animal (usually a rat) during a scan and correcting the PET data post-acquisition according the measured motion, such that a reconstruction can be made free of motion artefacts [10, 11]. Such motion compensation (MC) approaches avoid the need for anaesthesia and minimise the stress of the animal since it is unrestrained (although the animal is often confined to a small space during the scan). Motion tracking can either be marker-based [10, 11], where a small marker is attached to the head of the animal and tracked by external cameras, or markerless, where the facial features of the animal are identified and tracked [12]. These approaches have been shown to produce reconstructions of comparable quality to those of standard (anaesthetised) scans and thus show the greatest promise for investigating the effect of anaesthetics. In [13], a preliminary study was presented on tracer kinetics in a conscious and unrestrained rat, but otherwise, to the best of our knowledge, no other studies have been reported using this approach.

In this work, we report on a study of fully conscious, tube-bound, but unrestrained, rats undergoing dynamic scans to evaluate the effect of the anaesthesia induced by isoflurane on ^18^F-FDG uptake in the brain. An external stereo-optical system is used to track a marker attached to the rat’s head [11, 14], and a list-mode based motion compensation reconstruction is then performed to achieve a reconstruction free of motion artefacts [15].

This investigation was chiefly conducted as a proof-of-principle study, and therefore we made use of ^18^F-FDG since the effect of isoflurane on this tracer is largely understood [2, 4]. We aim to confirm the results found by these previous studies, to contribute to the understanding of the effect of isoflurane by scanning dynamically from the time of tracer infusion, and to demonstrate the efficacy of motion compensation in such studies. In the future, we will conduct similar studies on tracers where the effect of the anaesthetic is not as well understood and where a reference tissue model exists such that kinetic modelling can be performed without requiring arterial blood sampling.

## Methods

### Hardware

**Fig. 1:**
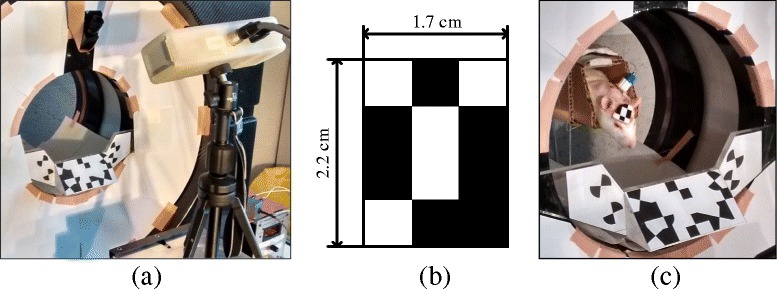
**a** The MicronTracker in front of the microPET scanner. The large marker attached to the front of the scanner is the reference marker used to aid in transforming from the MT coordinates to the microPET coordinates. **b** The head marker used in rat studies. **c** An unrestrained rat with an attached head marker inside a tube within the microPET. The catheter port can be seen between its shoulders. Figure reproduced with permission of IOP Publishing from [14]. Ⓒ Institute of Physics and Engineering in Medicine. All rights reserved

PET measurements were performed using the microPET Focus 220 small animal PET scanner (Preclinical Solutions, Siemens Healthineers, Knoxville, TN, USA). To track the motion, the MicronTracker Sx60 (ClaroNav Inc., Toronto, Canada) was used, as in [11]. The MicronTracker (MT) is a stereo-optical system which tracks preregistered planar markers, deriving the 6 degrees-of-freedom (i.e. the marker pose) from two simultaneously acquired but spatially offset images. It was used to track a small marker (2.2×1.7 cm) attached to the rat’s head, at a frequency of 25–30 Hz. The experimental setup is shown in Fig. 1.

#### Spatial calibration

Since the PET and MT coordinate systems are not necessarily aligned to each other, the transformation matrix to convert the recorded motion data into PET coordinates must be determined. A similar procedure to that described in [16, 17] was used: a radioactive point source is placed on the origin of an MT marker and scanned and tracked at various positions within the PET. The PET data were reconstructed using the commercial software of the scanner. By comparing the MT data with the PET reconstructions, it is possible to determine a suitable transformation between the coordinate systems [18]. A reference marker attached to the PET gantry (see Fig. 1) allows us to use the same calibration on different scan days even if the MT has been moved.

#### Temporal calibration

As each motion data point is measured a pulse is sent to the PET gate input to cause a tag to be inserted into the list-mode stream. Aligning the recorded motion data with the gate tags allows for the temporal calibration of the two data sets.

In previous work, we have optimised the experimental setup parameters for the MT and microPET [14], and the same parameters were utilised for this study. Our hardware and software for MC have been quantitatively validated using phantom measurements.

### Experiment protocol

All animal experiments were approved by the bioethical committee of the KU Leuven and performed in accordance with the European Communities Council Directive (86/609/EEC).

Five female Wistar rats were used (200–281 g), designated as “A–E”, three of which had repeat studies (indicated by subscripts in their designations), yielding eight data sets in total. Seven to ten days before the start of the study, a surgery was performed on each rat to insert a catheter into the femoral vein, with an access port protruding dorsally between the scapulae^1^. Before the surgery, medetomidine (0.3 mg/kg) and ketamine (60 mg/kg) were administered to the rats via an intraperitoneal injection. Following this, the rats were put on a 3-day course of antibiotics and analgesics. After recovery, each rat was then acclimatised to having the marker attached to its head, as well as being unrestrained inside a tube in the scanner, over a period of 3 days leading up to the scan for 30 min initially and increasing to 60 min. Since the rat was not restrained, it was possible for it to exit the tube onto the scanner gantry since the tube is approximately 10 cm above the scanner bore. If this occurred, the rat was then manually placed back in the tube and made fewer attempts to exit the tube as the days progressed. During scans, three of the rats did exit the tube once and were replaced in the tube immediately, but since their head motion was being tracked this had no effect on the final reconstruction, except for a short period where no PET data were acquired, which is automatically corrected for during reconstruction as explained in the “Reconstruction” section.

On the first day of the acclimatisation (3 days before the scan), the rats were anaesthetised with isoflurane for approximately 15 min to have the fur on their forehead removed for better marker attachment. The marker was attached to the rat’s forehead using “superglue”, which bonds and dries rapidly, but does not irritate the skin of the rat, thereby minimising discomfort, and which comes off by itself after several days. The marker and tube can be seen in Fig. 1 c. Whenever used, isoflurane was administered with a concentration of 2.5% in 2 L/min O_2_.

**Fig. 2:**
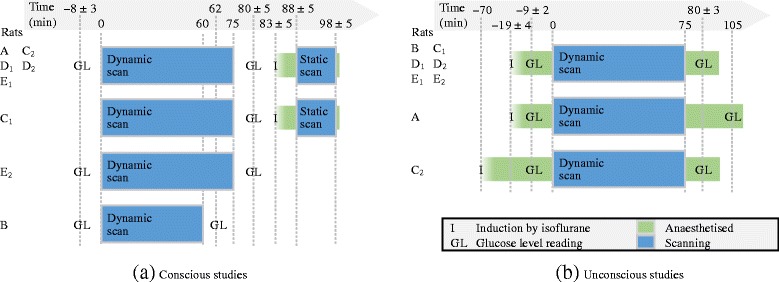
The timeline of the experimental protocol for the **a** conscious and **b** unconscious studies, for each rat. Time 0 is when the tracer was infused and the scan was started. For the time points with some spread, the mean ± standard deviation is quoted. Most rats followed the ideal protocol (first line), but there were some deviations for rats A, C_1_, C_2_, E_2_, and B, as can be seen

A schematic of the experimental protocol is shown in Fig. 2; a pre-determined protocol was used for the rats, but there were some deviations (as can be seen in Fig. 2) due to unforeseen circumstances beyond our control, but nonetheless these did not invalidate any of the data sets.

It is known that there are many factors which can affect FDG uptake [19–21] such as the blood glucose level, muscle usage, tracer administration route, body temperature, and stress, etc., and so where possible these were minimised or kept consistent across the rats. Preceding the scans the rats were fasted overnight for 12–15 h, with free access to water [22]. The rats were scanned while conscious for 60–75 min, starting from the tracer infusion. The tracer was infused via the intra-femoral vein catheter with 20–30 MBq of ^18^F-FDG in a total injected volume of 0.8 mL (including saline for flushing) using an infusion pump over 25 s. Immediately following this scan, the rats were anaesthetised with isoflurane and scanned for an additional 10 min while unconscious, to provide a validation of the MC reconstruction of the last frame of the conscious scan since the activity distribution in these reconstructions should be very similar. Two to three days after the conscious scan, the rats were again fasted and scanned with the same procedure, but while anaesthetised with isoflurane, which was administered before the tracer infusion and constantly throughout the scan. During all unconscious scans, the rats were placed on a heated mat to maintain body temperature. As suggested in [22], the blood glucose level of each rat was measured before and after each scan in duplicate using a drop of venal blood from a tail prick, with the GlucoCard Memory 2 meter (A. Menarini Diagnostics S.r.l., Florence, Italy), with exceptions being rat C_1_ where no reading was taken before the conscious scan, and rats C_2_, D_1_, and E_1_ where the readings were not taken in duplicate for the conscious scans.

While rat stress is a difficult factor to quantify, a similar setup was used by Fulton et al. [23] where body temperature was measured as a stress indicator using peritoneally implanted temperature sensors; Fulton et al. found that the body temperature did not elevate to levels indicative of stress while the rats were in the tube for up to 60 min. In our experiments, the rats exhibited no observable behavioural signs of stress: they engaged in grooming, resting, and exploring their (limited) surroundings.

### Reconstruction

After correcting each of the list-mode events’ endpoints according to the recorded motion, the reconstructions were performed using list-mode ordered subsets expectation-maximisation (OSEM) [24–27], using 10 iterations and 10 subsets, and a pixel size of [0.475, 0.475, 0.798] mm. The resolution was modelled using an image-based convolution [28] with a Gaussian kernel with a FWHM of 1.3 mm [29]. An attenuation map was constructed by extracting the body contour of the rat from a preliminary reconstruction (without attenuation correction) and filling it with the attenuation coefficient of soft tissue, ignoring the skull since it is very thin [30]. As suggested by [15], the data were pre-corrected for attenuation, and the sensitivity image was calculated as a weighted average of all poses. By analysing a reconstruction of an unconscious rat scan, it was established that the effect of scatter on the brain was less than 2.5%, and of randoms was around 1%, which was sufficiently small to ignore in the reconstructions.

If the rat temporarily moved out of the scanner field-of-view (such as in the few occurrences where the rat exited the tube), and whenever motion data was not available because, for example, the rat moved its head to an orientation where the marker could not be seen by the tracker, then the time-averaged sensitivity image would automatically have a zero contribution from those time points and thus would automatically scale the reconstruction to correct for these periods. In the latter case, any PET data acquired during these periods were ignored.

The rats were observed to touch the marker intermittently 1–4 times during the scans, and four rats managed to move the marker and cause some relative motion between the marker and the brain, which resulted in inter- and intra-frame motion in the reconstructions. Inter-frame motion was corrected for by frame-by-frame registration (after selecting a reference frame and ignoring early frames with low uptake). While this was necessary in only four of the studies (in the worst case resulting in a shift of about 3 mm within the brain over two frames), it was performed by default. Intra-frame motion of the marker relative to the brain would result in residual motion blur in the reconstructed image.

The unconscious and conscious scan reconstructions were aligned by an affine registration to the Johnson rat brain atlas [31], which was used to delineate regions-of-interest (ROIs). Since no arterial blood sampling could be performed on the conscious rats, and FDG has no reference tissue model in the rat brain, no input function could be derived and therefore the tracer kinetics could not be modelled.

### Data analysis

After correcting for deadtime and decay, the standard uptake values (SUVs) were calculated for each reconstruction using,

1 $$\begin{array}{@{}rcl@{}} \text{SUV}_{j} = \Lambda_{j} \frac{w}{d}, \end{array} $$

where *Λ*_*j*_ is the reconstructed activity concentration in pixel *j*, *w* is the rat’s body weight, and *d* is the injected dose.

Due to the variability in the glucose levels (as discussed in the “Blood glucose level” section), the data analysis was performed by calculating the ratio of various ROIs to the whole brain average. Such ratios are thus not affected by the scaling by the glucose level and yield information regarding how the regional distribution of the tracer was affected by the isoflurane. The ratios were calculated using

2 $$\begin{array}{@{}rcl@{}} R_{rik} = \frac{\bar{\Lambda}_{rik}}{\bar{\Lambda}_{0ik}},  \end{array} $$

where *r*, *i*, and *k* are the ROI, frame, and rat indices, respectively, $\bar {\Lambda }_{{rik}}$ is the mean value of the pixels inside the ROI *r*, and where *r*=0 indicates the whole brain ROI. The ROIs considered were the cortex, cerebellum, diencephalon, striata, vestibular nuclei, and inferior colliculi, shown in Fig. 6 a, b. The mean ratios across the rats are then denoted by $\bar {R}_{ri}$.

Since each rat was scanned with and without the anaesthetic they can be considered to be their own controls. A paired *t*-test between the unconscious and conscious scans was conducted on the ratios of six ROIs under consideration (using a Bonferroni correction factor of 6), at equilibrium, i.e. the sum of the last five frames spanning from 38 to 75 min of post-injection.

## Results

### Blood glucose level

**Fig. 3:**
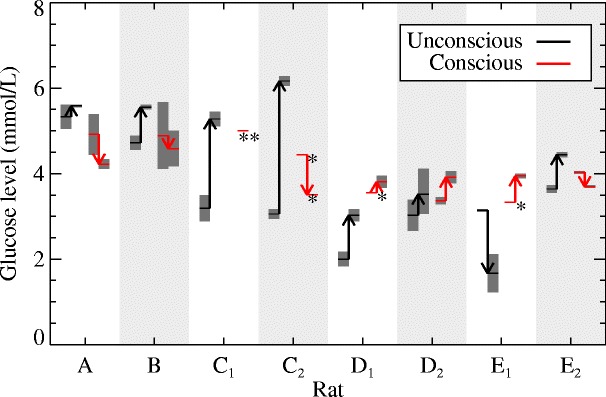
Blood glucose level measurements for each rat at the time of the unconscious (*black*) and conscious (*red*) scans. For each rat, the *arrows* start at the measurements taken before the scan and end at those taken after the scan. Most measurements were taken in duplicate, the extent of which are indicated by the *dark grey bars*. *Only a single measurement was taken. **No measurement was taken before the scan

The blood glucose measurements taken before and after each of the scans have been summarised in Fig. 3. For the conscious scans, the glucose level was relatively constant from before to after the scan across the rats, falling within a range of [ 3.3,5.0] mmol/L. During the unconscious scans, on the other hand, the glucose levels were often very different from before to after the scan and exhibited a greater range across the rats, falling within [ 1.7,6.2] mmol/L. For all unconscious scans, except E_1_, the glucose level rose, some substantially, from before to after the scan, while for E_1_ it dropped. This indicates an effect of the anaesthetic on the glucose level. Significant responses of the glucose level to isoflurane have been reported by other groups; an increase in the blood glucose level in the brain was observed for adult rats in [32, 33] after 60 to 75 min of isoflurane, while in neonatal mice a decrease in the glucose level was observed in [34]. An increase in glucose level was also observed in humans administered with isoflurane, and it is thought that this may be due to a decreased insulin response [35]. These effects all suggest that glucose regulation is affected by isoflurane, which is a further confounding factor in studies involving isoflurane.

Due to this variation of the glucose level, it was not evident which measurement (before, after, or the mean) should be used to normalise the reconstructions, as suggested in [22]. Therefore, it was decided to analyse relative changes in the uptake of FDG in the rat brain, which are independent of the glucose level, for the various ROIs, as shown in Eq. (2). In addition, however, a comparison of the whole brain average in the conscious and unconscious scans was also conducted.

### Reconstructions

**Fig. 4:**
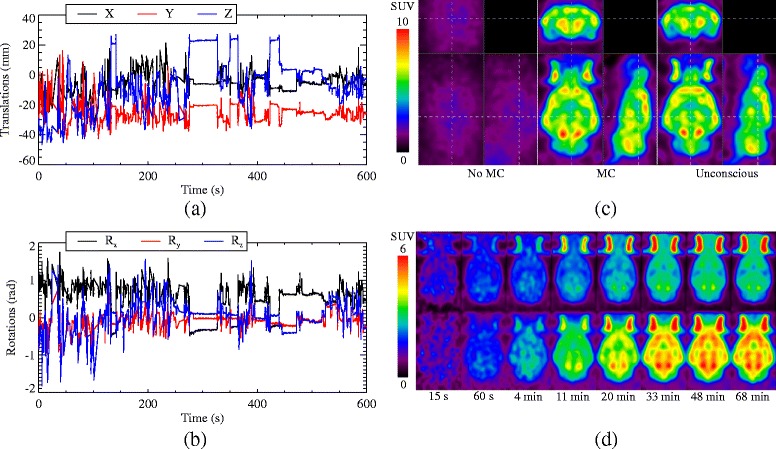
The translations (**a**) and rotations (**b**) of a rat’s head during a 10-min conscious scan. **c** Non-MC (*left*) and MC (*middle*) reconstructions of the conscious scan, which started 60 min after tracer injection, along with a reconstruction (*right*) of an unconscious scan of 10 min taken of the same rat, 91 min after tracer injection. The *dashed lines* indicate where the image slices are located. **d** A selection of dynamic frames from the unconscious (*top*) and conscious (*bottom*) scan reconstructions, averaged over all eight data sets. The times below the frames indicate the frame start time since the tracer injection

The measured motion of a conscious rat during a 10 minute scan is shown in Fig. 4 a, b, with the MC reconstruction in Fig. 4 c along with the subsequent unconscious scan reconstruction. Much of the resolution lost due to the motion has been recovered, and the activity distribution is very similar between the conscious and unconscious scan reconstructions. Since the rat moved significantly during the scan, the activity concentration is much lower in the non-MC reconstruction; however, the total activity is approximately equal to the MC reconstruction, as expected.

**Fig. 5:**
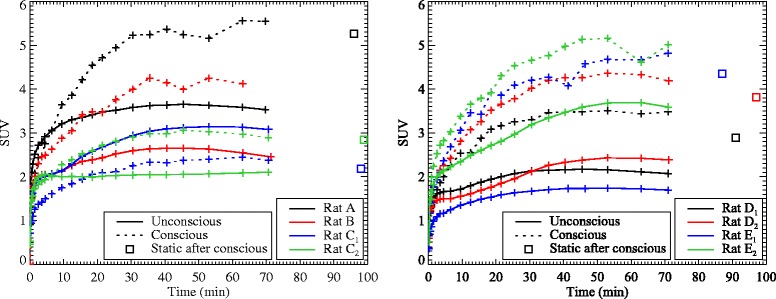
TACs for all eight data sets for the whole brain ROI, for the conscious (*dotted lines*) and unconscious (*solid lines*) scans, as well as the static scan with anaesthesia following the conscious scan (*square points*). The data sets are divided over two plots for clarity. For rats B and D_2_, the static scan data was not available. No *error bars* are plotted since the ROI is non-uniform

A selection of the dynamic frames for the average of the eight data sets is shown in Fig. 4 d for the conscious and unconscious scans. In Fig. 5, the time activity curves (TACs) for the whole brain region for all eight data sets is shown. The unconscious, static scans taken after the conscious scans are in good agreement with the last frames of the conscious scans, possibly showing some degree of washout [36], a trend which is observable in the last few frames of the dynamic scan.

To investigate whether there was a correlation between the SUVs and glucose levels, a Pearson product-moment correlation analysis was conducted on the SUVs at equilibrium (i.e. the sum of the last five frames) versus both the pre- and post-scan glucose levels. In the conscious case, the correlation coefficients were −0.19 and −0.28 for the pre- and post-scan glucose level, respectively (indicating no correlation) and in the unconscious case they were 0.62 and 0.53 for the pre- and post-scan glucose level, respectively. However, the *p* values for these fits were higher than 0.05 and therefore there is insufficient evidence to conclude that a correlation exists between the SUVs and the glucose levels.

A paired *t* test was performed on the SUVs at equilibrium for the whole brain region to determine whether the uptake in that ROI was significantly different between the conscious and unconscious scans. The change from unconscious to the conscious scan had a mean of 59±52%, and the *p* value of the test was 0.007, which is significant at the *p*<0.05 level.

**Fig. 6:**
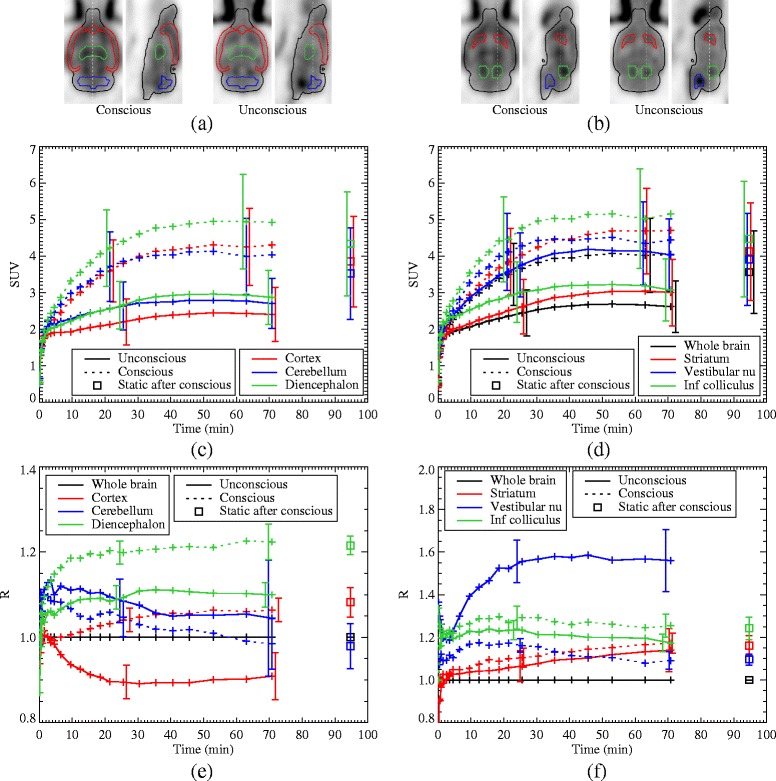
**a**, **b** The ROIs under consideration are indicated on the last frame from the average of the eight reconstructions. **c**, **d** TACs for various ROIs for the average of the eight data sets, for the conscious (*dotted lines*) and unconscious (*solid lines*) scans, as well as the static scan with anaesthesia following the conscious scan (*square points*). The *overlapping error bars* are staggered for clarity. **e**, **f** The ratio of various ROIs to the whole brain average, averaged over the eight data sets. In all plots, the *error bars* are the standard deviations across the data sets and are only shown in two representative locations for clarity

The TACs for the six ROIs for the average of the eight data sets are shown in Fig. 6 c, d. Plots of the ratios between each of the ROIs and the whole brain average are shown in Fig. 6 e, f. Again, the unconscious, static scans show good agreement with the last frames of the conscious scan. The results of the paired *t* test analysis on the ROI ratios at equilibrium are summarised in Table 1. For the conscious scans, the relative changes in the cortex (18.2±7.1%), vestibular nuclei (−29.5±6.2%), diencephalon (10.3±4.7%), and inferior colliculi (5.3±3.4%) compared to the unconscious scan were significant with *p*<0.05 (i.e. *p*<0.008 after the Bonferroni correction). No significant changes (at the same *p* level) were observed in the cerebellum and striata.

**Table 1:** Average ratio between ROIs and whole brain at equilibrium

	Uncon.	Con.	Change ± SD (%)	*p*	Sig.
Cortex	0.90	1.06	18.2±7.1	7.4×10^−5^	Yes
Vestibular nu.	1.57	1.10	−29.5±6.2	2.7×10^−5^	Yes
Diencephalon	1.10	1.22	10.3±4.7	0.0004	Yes
Inf. colliculi	1.20	1.26	5.3±3.4	0.0030	Yes
Cerebellum	1.05	1.00	−3.7±8.9	0.2322	No
Striata	1.12	1.15	3.7±8.0	0.2920	No

## Discussion

In this study, we have demonstrated the efficacy of using a motion compensation technique to study fully conscious and unrestrained rats in a dynamic study. We investigated the effect of the anaesthetic isoflurane on the uptake of FDG in the rat brain, and a clear and significant impact of the isoflurane on the regional distribution of FDG uptake in the rat brain was observed.

From the blood glucose level measurements before and after the scans, we observed a large variability in the glucose levels of the unconscious rats, and a substantial change in the glucose level from before to after the scan, which is in agreement with other reported studies [32, 33]. In one test-retest (for rat E), the behaviour of the glucose level was not consistent. No statistical correlation was found between the SUVs at equilibrium and the glucose levels. Therefore, if a glucose correction is to be performed on the SUVs when using isoflurane, then this variability must be taken into consideration. On the other hand, the glucose levels of the conscious rats were much more constant from before to after the scan and between different rats.

Even though the conscious rats exhibited a large range of motion during the scan, the data were successfully compensated for motion and much of the resolution lost was regained. All of the rats tolerated the acclimatisation and scanning very well, with only three actually exiting the tube during a scan (before being placed back in the tube) which did not adversely affect the reconstructions. On occasion, some rats did try to remove the marker during the scan, thus possibly inducing relative motion between the marker and the brain. This resulted in some inter- and intra-frame motion, the former of which was corrected for by frame-by-frame registration and the latter possibly causing some residual motion blur in the reconstructions. The motion compensation was verified by comparing the last frame of the conscious scan to the subsequent, static, unconscious scan.

Since no blood input function could be measured for the conscious rats, and FDG has no reference tissue model in rats, a full kinetic analysis could not be performed. Nonetheless, from the results of the dynamic scans presented in Figs. 5 and 6, it is clear that the isoflurane has a substantial impact on the tracer uptake. A paired *t* test confirmed that there was a significant difference between the whole brain SUVs at equilibrium in the conscious and unconscious scans (*p*<0.05). Furthermore, an analysis of the regional distributions within the brain showed a significant difference in the cortex, diencephalon, vestibular nuclei, and inferior colliculi between the conscious and unconscious scans (*p*<0.05). Previously, Matsumura et al. [4] conducted a study into the effect of various anaesthetics (including isoflurane) on FDG uptake in rats. They acquired static images of anaesthetised rats after allowing for a conscious uptake period and found similar regional differences to those found in this study: a decrease in the frontal cortex (although with a smaller magnitude increase in the posterior cerebral cortex), a decrease in the thalamus (a substructure of the diencephalon), and no change in the cerebellar cortex (a substructure of the cerebellum) and the striata. Their approach, unlike that presented in this work, cannot be used to investigate the effect of anaesthesia on tracer kinetics, which requires dynamic scanning from the time ofinjection.

In the unconscious scans, there was a strong contrast between the vestibular nuclei and the whole brain, while this contrast was not observed in the conscious scans. In [37], Gupta et al. investigated the effect of isoflurane on the vomiting reflex. They used musk shrews instead of rats since the latter do not exhibit a vomiting reflex (however, the rat brain may nonetheless respond similarly to isoflurane). They found that isoflurane induced a high c-Fos cell count (a protein related to neuronal activity) in the vestibular nuclei, where vestibular inputs are integrated with inputs from other sensory systems and the cerebellum for the perception of balance. High neuronal activity in the vestibular nuclei would likely be accompanied by an increased glucose uptake, explaining the high FDG uptake in the vestibular nuclei in the unconscious scans compared to the whole brain average.

## Conclusions

The effect of isoflurane on the uptake of FDG in the rat brain has been investigated using a motion compensation technique to scan fully conscious and unrestrained rats dynamically from the time of tracer infusion. Although relatively few animals were studied, since each animal could be considered to be its own control, our results show a clear and significant impact of the isoflurane on the regional distribution of FDG in the rat brain. A significant difference was observed in the cortex, diencephalon, vestibular nuclei, and inferior colliculi, while no significant effect was observed in the cerebellum and striata. A strong variability was observed in the blood glucose levels of the anaesthetised rats between scans, and from before to after the scan, which suggests that this could be a further confounding factor of isoflurane on FDG studies. This work demonstrates that if isoflurane is to be used in FDG studies, these confounding factors should be recognised and taken into account.

In the future, we will perform similar studies on other common PET tracers, especially those with a reference tissue model where kinetic modelling can be performed, such as raclopride or fallypride. For many such tracers, the influence of anaesthesia is not completely understood.

## Endnote

^1^ One rat (not counted in the eight data sets) was excluded from the study after it was established (postmortem) that the inserted catheter had come out of the vein.
